# All trans-retinoic acid analogs promote cancer cell apoptosis through non-genomic Crabp1 mediating ERK1/2 phosphorylation

**DOI:** 10.1038/srep22396

**Published:** 2016-03-03

**Authors:** Shawna D. Persaud, Sung Wook Park, Mari Ishigami-Yuasa, Naoko Koyano-Nakagawa, Hiroyuki Kagechika, Li-Na Wei

**Affiliations:** 1 Department of Pharmacology University of Minnesota, Minneapolis, MN 55455, USA; 2 Tokyo Medical and Dental University (TMDU), Institute of Biomaterials and Bioengineering, 2-3-10 Kanda-Surugadai, Chiyoda-ku, Tokyo 101-0062, JAPAN; 3 Department of Medicine, University of Minnesota, MN 55455, USA.

## Abstract

All *trans* retinoic acid (atRA) is one of the most potent therapeutic agents, but extensive toxicity caused by nuclear RA receptors (RARs) limits its clinical application in treating cancer. AtRA also exerts non-genomic activities for which the mechanism remains poorly understood. We determine that cellular retinoic acid binding protein 1 (Crabp1) mediates the non-genomic activity of atRA, and identify two compounds as the ligands of Crabp1 to rapidly and RAR-independently activate extracellular signal regulated kinase 1/2 (ERK1/2). Non-canonically activated ERK activates protein phosphatase 2A (PP2A) and lengthens cell cycle duration in embryonic stem cells (ESC). This is abolished in Crabp1-null ESCs. Re-expressing Crabp1 in Crabp1-negative cancer cells also sensitizes their apoptotic induction by atRA. This study reveals a physiological relevance of the non-genomic action of atRA, mediated by Crabp1, in modulating cell cycle progression and apoptosis induction, and provides a new cancer therapeutic strategy whereby compounds specifically targeting Crabp1 can modulate cell cycle and cancer cell apoptosis in a RAR-independent fashion, thereby avoiding atRA’s toxicity caused by its genomic effects.

All-*trans* retinoic acid (atRA), the active ingredient of Vitamin A, affects diverse biological processes including development, growth, immune function, neural function, reproduction, and vision. Like other steroid hormones, atRA elicits genomic action via its nuclear receptors retinoic acid receptors (RARs) and non-genomic action. The non-genomic mediator of atRA and its mechanism of action remain poorly understood. For estrogens, studies reported membrane-associated estrogen receptor (ER) as the principal endogenous mediator for their non-genomic action^1^,^2^,^3^. For thyroid hormones, a study reported a yet-to-be identified membrane-localized receptor for its non-genomic action modulating nitrate oxide synthase, guanylyl cyclase and protein kinase GII^4^. Interestingly, glucocorticoids can involve a membrane-bound glucocorticoid receptor (GR) and a non-classical membrane GR that is a glycoprotein^5^ for non-genomic activities.

Typical genomic action of steroid hormones occurs after a time lag of several hours, and alters target gene expression lasting for an extended period of time^6^. On the contrary, non-genomic steroid actions such as thyroid hormone occur much faster, ranging from minutes to seconds, and can affect multiple signaling pathways^6^. However, the physiological implication of these non-genomic actions has been debated. Most critically, a lack of specific ligands that can selectively elicit the “non-genomic” action of these hormones without acting on their nuclear receptors has prevented a validation of these studies and hindered the progress in the field. For atRA, non-genomic action has been reported in multiple studies^7^, such as activating PKCα^8^, binding to membrane RAR to affect neuron spine formation^9^, and rapidly activating extracellular signal regulated kinase 1/2 (ERK1/2)^10^,^11^. ERK activation by atRA has been most widely detected in different experimental model systems. This current study establishes a physiological role for Crabp1 in mediating the non-genomic activities of atRA, and identifies specific ligands for Crabp1 that can elicit atRA’s non-genomic actions without involving RARs.

The non-genomic effects of atRA are particularly interesting in a stem cell context such as embryonic stem cells (ESCs) and cancer cells. Maintaining their self-renewal requires a tight control over cell cycle progression that ultimately governs cell proliferation, differentiation, senescence and apoptosis^12^. It is known that during differentiation protein phosphatase 2A (PP2A) activity gradually increases; and inhibiting PP2A promotes ESC self-renewal^12^. Interestingly, in this current study we identify PP2A as a target of non-genomic ERK1/2 activation, elicited by holo-Crabp1, in ESC.

From a translational point of view, atRA can be a potent therapeutic for various diseases because of its anti-proliferative, anti-oxidative, pro-apoptotic, and differentiation-inducing activities. It has been most successful in treating acute promyelocytic leukemia^13^. In animal models of skin, oral, lung, breast, bladder, ovarian, and prostate cancers, atRA has also been found to suppress carcinogenesis^14^,^15^,^16^,^17^,^18^,^19^. In various human clinical trials to prevent oral, head and neck, non-melanoma skin cancers, breast, cervical, and hepatocellular carcinoma, retinoids were a highly promising category of compounds that have been investigated^20^. However, toxic side effects and retinoic acid syndrome have drastically limited clinical use of these compounds. These toxicities are mainly attributed to retinoids’ canonical, genomic actions through binding to RARs^21^. Furthermore, at therapeutic doses atRA loses efficacy as RA resistance develops. RA resistance currently is attributed to RAR action and hinders therapeutic potential of retinoid compounds^22^,^23^. An important question is, whether it is possible to elicit certain desirable effects of retinoids without activating the wide spectrum RAR-mediated genomic actions. To this end, this study reports two compounds that act specifically by binding to Crabp1 to elicit activity mimicking atRA’s non-genomic (RAR-independent) activity to augment ESC cell cycle progression and stimulate cancer cell apoptosis. For a proof-of-concept, the current study further demonstrates a strategy using these compounds to enhance apoptosis specifically in Crabp1-positive cancer cells. These results implicate a therapeutic strategy exploiting a new signaling pathway that can avoid deleterious toxicity caused by conventional retinoid therapy.

## Results

### Ligands for Crabp1 induce rapid, RAR-independent activation of ERK1/2

**Figure 1:**
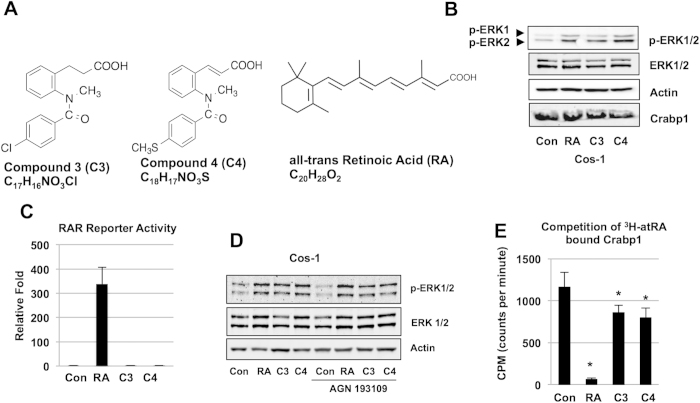
RAR-independent, rapid activation of ERK1/2 by compounds 3 and 4. (**A**) Structure of compounds. (**B**) Activation of ERK1/2 in Cos-1 assessed by western blot analyses under 100 nM for 30 min. Upper band depicts ERK1 (44 kDa) and lower band depicts ERK2 (42 kDa). (**C**) Compounds 3 and 4 do not activate RAR activity as detected by RAR reporter assay performed in Cos-1 cells treated with compounds at 250 nM for 24 hrs. (**D**) Pan-RAR antagonist AGN 193109 (100 nM, 1 hr pretreatment) fails to block rapid ERK activation in Cos-1 cells. Data (**B**–**D**) are representative of at least 3 independent experiments. (**E**) *In vitro* competition assay to displace ^3^H-atRA from Crabp1 by atRA, C3, and C4. Data is displayed as counts per minute (CPM). Asterisk shows significance: RA (p < 0.001), C3 (p = 0.023) and C4 (p = 0.014) versus control, and mean ± S.E.M (n = 4).

Retinoids are structurally comprised of a bulky hydrophobic region, a terminal polar functional group (carboxyl group or its bioisosteres), and the linker between them^24^. We screened compounds of a structural similarity with retinoids and bearing a hydrophobic region and a terminal carboxyl group or its bioisostere, and excluded those acting through RARs. Through these screenings, we identified compounds 3 and 4. Compound 3 has a chemical formula of C_17_H_16_NO_3_Cl, and compound 4 has a chemical formula of C_18_H_17_NO_3_S (Fig. 1A). Compounds and atRA elicit rapid (<30 min) ERK1/2 activation in cells with abundant Crabp1 (Fig. 1B) without acting on atRA’s genomic mediators RARs using a conventional nuclear RAR reporter assay (Fig. 1C). The RAR-independent action was further confirmed because pan-RAR antagonist AGN 193109 pre-treatment failed to inhibit activation of ERK1/2 by these compounds (Fig. 1D). We then confirmed that these compounds could directly bind Crabp1 (Fig. 1E) using competitive *in vitro* binding assays. Compounds 3 (p = 0.023) and 4 (p = 0.014) were able to significantly displace ^3^H-atRA from Crabp1. These data show direct binding of these compounds to Crabp1, albeit at a lower affinity than that of atRA. Their specificity to Crabp1 was further validated using Crabp1 mutant proteins (see later).

### Non-genomic ERK1/2 activation stimulates PP2A and expands G1 cell cycle phase duration in ESC, which is abolished in Crabp1-null ESC

**Figure 2:**
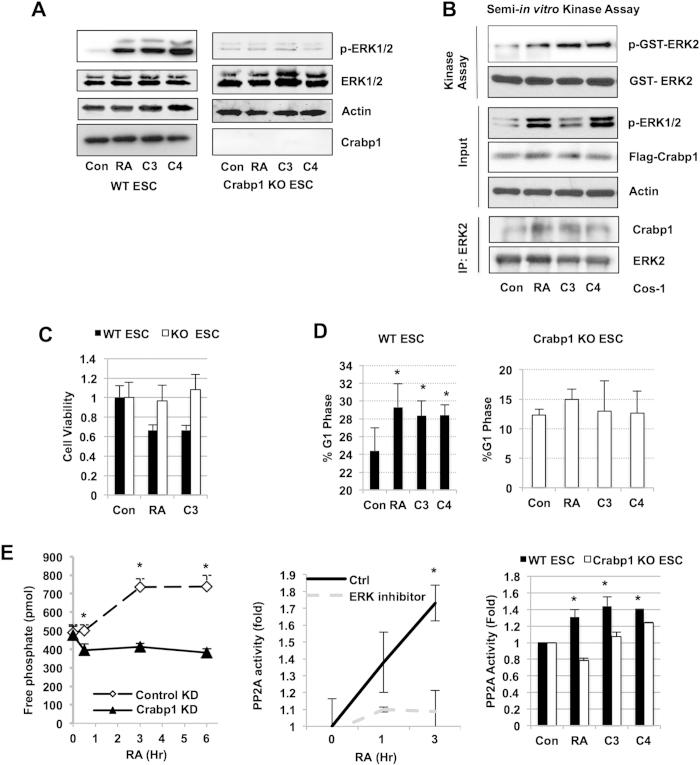
Crabp1-ERK1/2 activation by compounds 3 and 4 stimulates PP2A in ESC. (**A**) ERK activation elicited by RA and compounds 3 and 4 for 30 min is abolished in Crabp1 KO ESC. (**B**) Semi-*in vitro* kinase assay. Partially immuno-purified Crabp1 complex (bottom) activates recombinant ERK1/2 *in vitro* under 100 nM RA, C3, and C4 treatment for 30 min. (**C**) RA and Compound 3 decrease cell viability as detected by MTT assay with 100 nM treatments for 24 hrs. (**D**) G1 phase expansion by RA and compounds 3 and 4 in WT ESC but not in Crabp1 KO ESC. Cells were treated with RA and compounds 3 and 4 at 100 nM for 12 hrs before flow cytometric analysis. Asterisk shows significance: RA (p = 0.05), C3 (p = 0.05) and C4 (p = 0.04) versus control, and mean ± S.E.M (n = 4). (**E**) WT ESCs were transfected with scrambled (control) and *siCrabp1* (Crabp1 KD) followed by RA treatment (100 nM). RA-induced phosphatase activity (detected for free phosphate) is blocked by Crabp1 knockdown. Asterisk shows significance: 30min (p = 0.01), 3hr (p = 0.002), and 6hr (p = 0.004) versus WT ESC (left). RA-stimulated PP2A activation is blocked by ERK1/2 inhibitor, FR180204; 3 hr (p = 0.03) versus vehicle (center). RA, compounds 3 and 4 stimulate PP2A activity after 100 nM treatment for 3 hrs in WT ESC but not in Crabp1 KO ESC. RA (p < 0.001), C3 (p = 0.017), and C4 (p = 0.001) versus control (right). Data (**A**–**E**) are representative of at least 3 independent experiments.

To validate the functional role of Crabp1 in mediating this pathway, we generated Crabp1 knockout (KO) ESC, and confirmed that atRA, compounds 3 or 4 could no longer rapidly activate ERK in Crabp1 KO ESC (Fig. 2A, left) as compared to WT ESC’s (Fig. 2A, right). To directly validate the functional role of these compounds in activating ERK1/2 through Crabp1, we performed an *in vitro* kinase assay that detected enzymatic activation of ERK1/2 using partially purified holo-Crabp1 complexes from cells stimulated with either atRA, compound 3 or compound 4. As shown in Fig. 2B, the Crabp1 complexes, purified under atRA, compounds 3 or 4 stimulation, were able to activate recombinant GST-ERK2 *in-vitro*, while the complex from control cells failed in this *in-vitro* reaction. These data show that the holo-Crabp1 complex, with atRA, compound 3 or compound 4, can directly activate ERK1/2 activity. Mechanistically, this suggests that Crabp1 may act as a scaffold to regulate ERK activity, as shown in co-association of Crabp1 with immunoprecipitated ERK2 (Fig. 2B, lower panel). We then found that compound 3 like atRA, decreased cell viability in WT ESC but had no effect in Crabp1 KO ESC as detected in MTT assay (Fig. 2C). Further, like RA, these compounds also expanded the G1 phase in WT ESC (from 24.4 to 29.3 percent for RA and to 28.3 percent and 28.4 percent for compounds 3 and 4, respectively) but not in Crabp1 KO ESC (Fig. 2D). Interestingly, we found atRA rapidly elevated the level of protein phosphatase 2A (PP2A) activity that was abolished by silencing Crabp1 (Fig. 2E, left). ERK inhibition blocked elevation in PP2A activity (Fig. 2E, center), suggesting the non-genomic effect of atRA can be extended to PP2A that is important for cell cycle control. As an ultimate proof, we confirmed that atRA, and compounds 3 and 4 could no longer activate PP2A in Crabp1 KO ESCs (Fig. 2E, right).

### Non-genomic ERK1/2 activation is lost in Crabp1-negative cancer cells

**Figure 3:**
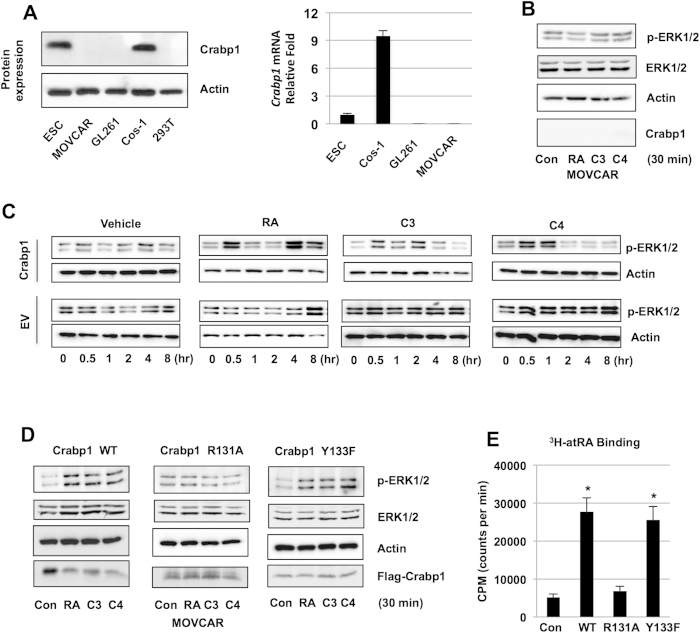
Crabp1-dependent activation of ERK1/2 by compounds 3 and 4 requires ligand binding. (**A**) Crabp1 expression assessed on protein (left) and mRNA (right) levels. (**B**) Western blot analysis. Compounds 3 and 4 fail to activate ERK1/2 in Crabp1-negative MOVCAR at 30 min, 100 nM. (**C**) Western blot analyses of kinetics of ERK1/2 activation by compounds (100 nM). MOVCAR cells re-expressing Crabp1 (upper panels) or empty vector (EV, lower panels) were treated with vehicle, RA, compound 3 or compound 4 for the indicated hrs. Early phase of ERK1/2 activation is detected in Crabp1 expressing cells. (**D**) Rapid ERK1/2 activation in MOVCAR re-expressing wild type or mock-mutated Crabp1 Y133F but no activation in cells re-expressing Crabp1 R131A, RA binding deficient mutant at 30 min, 100 nM treatments. (**E**) ^3^H-atRA ligand binding assay reveals RA binding to wild type and Crabp1 Y133F but no binding to Crabp1 R131A proteins. *p < 0.001 versus to control. Data is displayed as counts per minute (CPM). Data (**A**–**E**) are representative of at least 3 independent experiments.

**Figure 4:**
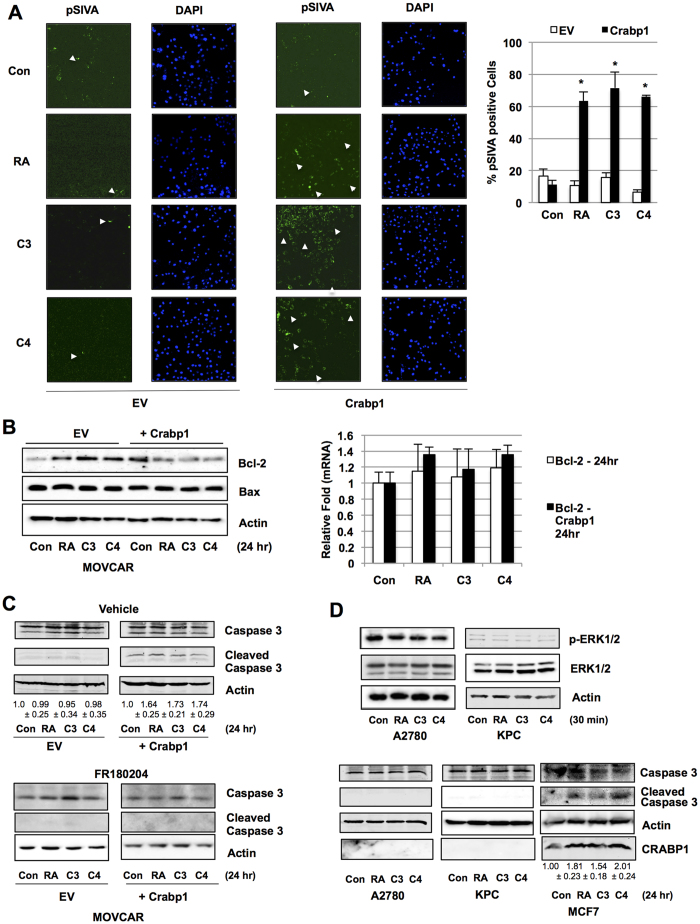
Crabp1-dependent induction of apoptosis by compounds 3 and 4. (**A**) Images showing apoptosis of Crabp1-positive MOVCAR cells (pSIVA staining, green). Cells were treated with 100 nM for 24 hrs. Quantitation of pSIVA positive cells shown on the right: *p < 0.001 versus control, n = 3, EV: empty vector. (**B**) Decrease in Bcl-2 protein by atRA, compound 3 or 4 (left), without altering mRNA expression (right). (**C**) Increase in cleaved caspase 3 (upper right), which is blocked by ERK1/2 inhibitor (below) in MOVCAR. This is abolished in the absence of Crabp1 (upper left, EV) under 100 nM RA, C3, and C4 treatment for 24 hrs. Quantification of cleaved caspase 3 is shown (upper, n = 4). (**D**) Compounds lose ability to induce ERK activity in human CRABP1 null cancer cell lines A2780 (ovarian) and KPC (pancreatic ductal carcinoma) after 100 nM, 30 min treatment (upper). Compounds are able to induce apoptosis after 24 hr treatment (lower) in CRABP1 positive MCF7 breast cancer cell line (lower right). Quantification of cleaved caspase 3 is shown for MCF-7. Data (**A**–**D**) are representative of at least 3 independent experiments.

Several genetic association studies have reported correlation of reduced Crabp1 expression in certain cancers/tumors^25^,^26^,^27^,^28^. As shown in Fig. 3A, Crabp1 expression was not detected in mouse ovarian cancer cell line (MOVCAR), glioma cell line GL261 or human embryonic kidney cell line 293T, but remained robust in Cos-1 and mouse ESC. However, MCF7 cancer cells do express CRABP1 (see later in Fig. 4D). Crabp1-negative cells may reflect either the lack of expression in certain cell populations that give rise to these cancer cells, or epigenetic changes of the original cells that give rise to these cancer cells. To test our hypothesis that Crabp1 is functionally involved in mediating ERK activation, we used MOVCAR cell (Crabp1-negative) to examine the effect of Crabp1 in mediating rapid ERK1/2 activation by atRA and compounds 3 and 4. As shown in Fig. 3B, without expression of Crabp1 in MOVCAR, atRA, compounds 3 and 4 failed to elicit early activation of ERK1/2. Crabp1 was then re-introduced into MOVCAR cells and kinetics of ERK1/2 activation by atRA and compounds 3 and 4 were analyzed on western blots (Fig. 3C). The results show that compound 3 activates ERK1/2 at 0.5–2 hr and compound 4 transiently at 0.5–1 hr, suggesting non-genomic activation (upper panels). Similar to what we reported in ESC and Cos-1 cells^10^, atRA biphasically activates ERK1/2 in MOVCAR cells re-expressing Crabp1 (upper panel). This rapid activation by atRA and compounds 3 and 4 was not detected in cells without Crabp1 (lower panels). These data show that in a cancer cell context, Crabp1 expression is critical for rapid ERK1/2 activation by atRA and compounds 3 and 4.

### Non-genomic ERK1/2 activation by compounds 3 and 4 is dependent on binding to Crabp1

Crabp1 is classically known for its role in binding and sequestering atRA in the cytoplasm to regulate intracellular atRA concentrations. Structural studies have provided insight into the interactions of atRA with the ligand-binding pocket of Crabp1^29^,^30^,^31^. Based upon these structural data, we previously generated atRA-binding mutant Crabp1 specifically at residues arginine 131 and tyrosine 133. Arginine R131 to alanine (R131A) mutation severely abrogated Crabp1 interaction with atRA, but tyrosine 133 mutation, such as Y133F, did not^32^. We thus used the R131A mutant Crabp1 to determine whether Crabp1’s ligand binding was required for activating ERK1/2. As shown in Fig. 3D, upon re-introducing R131A mutant Crabp1, atRA and compounds 3 and 4 remained unable to activate ERK1/2, whereas introducing the wild type Crabp1 successfully resumed ERK1/2 activation. As a control, the Y133F mutant Crabp1, which did not affect ligand binding^32^, could still elicit non-genomic activation of ERK1/2. These data show that, ligand binding of Crabp1, which requires R131, is critical for the non-genomic action of atRA, as well as ERK activation by Crabp1 ligands. Finally, we directly confirmed the ligand binding ability of purified Crabp1 mutant proteins as shown in Fig. 3E.

### Crabp1 ligands stimulate cancer cell apoptosis

The non-genomic activity of atRA/Crabp1 affects PP2A and modulates cell cycle progression in ESC (Fig. 2), which supports genetic association studies suggesting Crabp1 as a tumor suppressor. We thus monitored apoptosis in cancer cells with and without Crabp1 expression in pSIVA staining that detects surface phosphatidylserine^33^. As shown in Fig. 4A, in Crabp1-negative MOVCAR cells transfected with a control vector, neither atRA nor compounds 3 or 4 significantly changed pSIVA-positive cell populations. However, in MOVCAR cells expressing wild type Crabp1, atRA, compounds 3 and 4 each triggered a robust increase in pSIVA-positive cells populations (approximately 60–70%, right panel). This is supported by key apoptosis marker Bcl-2 (Fig. 4B). Note that while the level of pro-apoptotic protein Bax was not altered, the antiapoptotic Bcl-2 level was reduced, resulting in a higher ratio of Bax over Bcl-2 expression. This further suggests that holo-Crabp1 elicited apoptosis may target (reduce) the effectiveness of anti-apoptotic pathway. Very interestingly, mRNA levels of *Bcl-2* were unaffected; suggesting holo-Crabp1 post-transcriptionally affects Bcl-2 protein level. Consistently, in the presence of ERK1/2 inhibitor FR180204 (Fig. 4C), atRA, compound 3 or 4 could no longer induce cleavage of caspase 3. Given the ability of atRA and compounds 3 and 4 to induce apoptosis in animal cancer cell line context, we surveyed several human cell lines and found that CRABP1 expression was lost in human ovarian cancer cell line A2780 and human pancreatic ductal carcinoma cell line KPC. Interestingly, in these CRABP1 negative cell lines, atRA, compounds 3 and 4 failed to activate ERK1/2 in 30 minutes (Fig. 4D, upper panels), or induce cleavage of caspase 3 after 24 hr treatment. This is in contrast to CRABP1-positive human breast cancer cell line MCF-7 in which apoptosis was readily induced by atRA and compounds 3 and 4, as indicated by the presence of cleaved caspase 3.

## Discussion

In this study we determine the physiological role of Crabp1 in a stem cell context, which is to mediate atRA’s non-genomic signaling pathway in modulating cell cycle progression. We further identify specific ligands of Crabp1, which elicit activity mimicking the non-genomic activity of atRA without involving RARs. As a proof of concept, we examine these compounds, through binding to Crabp1, in enhancing Crabp1-positive cancer cell apoptosis. To this end, several genetic association studies have reported correlation of reduced Crabp1 expression in cancers/tumors. In serous ovarian adenocarcinomas and clear cell ovarian adenocarcinomas, reduced Crabp1 expression was tied to significantly poorer survival prognosis^28^. Epigenetic studies in which promoter methylation status of several genes in primary ovarian carcinomas and their *in vitro* models were assayed showed that the *Crabp1* gene promoter and the CpG islands of its coding region were hypermethylated – indicating a loss of Crabp1 expression^27^. Our earlier studies have also reported that DNA methylation on the *Crabp1* gene promoter is an important regulatory mechanism of its gene activity^34^,^35^. In esophageal squamous-cell carcinoma cells, absence of Crabp1 promoted cell growth^26^. Similar trends were found in colon cancer cell lines HCT116 and SW48 and in primary colorectal carcinomas^36^, as well as in papillary thyroid carcinomas^25^,^37^. This current study reveals a mechanism in which Crabp1 can play a role as a tumor suppressor, through non-canonical activation of ERK1/2 that then induces apoptotic pathways, likely through inhibiting anti-apoptosis, for cancer cell death. Importantly, these activities were abolished in Crabp1-negative cells. The identification of Crabp1 specific ligands, as well as its mechanism of action, sheds important insights into a potential new retinoid-based therapeutic strategy that can avoid toxicity associated with the genomic action mediated by RARs.

Multiple genes regulate the apoptotic pathway including *Bcl-2* and *Bax*. Bcl-2 exerts anti-apoptotic effects through a variety of mechanisms including inhibition of caspase activation by blocking cytochrome C release from the mitochondria. Bcl-2 binds to Bax to prevent its ability to induce permeabilization of the mitochondrial membranes that trigger cytochrome C release and subsequent caspase activation^38^. The ratio of these proteins is critical in determining the efficiency of apoptosis. A high level of Bax counteracts the anti-apoptotic effect of Bcl-2^39^, and a high Bcl-2/Bax ratio is associated with various subtypes of cancer that are highly resistant to chemotherapies^23^,^39^,^40^,^41^. In fact, several clinical trials are investigating the co-administration of compounds to enhance chemotherapeutic sensitivity in cancers with high resistance^42^,^43^. The ability of holo-Crabp1 to increase the Bax/Bcl-2 ratio also suggests a potential to sensitize resistant cancer cells to current therapies. Consistently, holo-Crabp1 also increases PP2A activity, which is suggested as a tumor suppressor^44^ that decreases Bcl-2 anti-apoptotic activity through direct dephosphorylation^44^,^45^,^46^.

Further development of Crabp1-selective ligands may prove efficacious in therapeutic application of retinoids. Compounds 3 and 4 elicit strong pro-apoptotic activity through binding to Crabp1. It is interesting to note that while these compounds do not bind to Crabp1 as tightly as atRA, they still are able to induce, almost as efficiently as atRA, non-canonical activation of ERK1/2. The fact that these compounds failed to elicit activities through mutant Crabp1 defective in atRA binding would suggest that occupying this ligand binding pocket is important for the activation of Crabp1 to elicit ERK activity. Structural studies may yield insight into how these molecules directly bind to Crabp1 and provide a basis for more rational drug design in the future.

## Materials and Methods

### Compounds

The Kagechika chemical compound library includes about 5,000 compounds that were synthesized in his laboratory for different purposes, including targeting nuclear receptors, in TMDU. Fifty compounds were selected from this library by the structural similarity with known retinoids, bearing some hydrophobic region and the terminal carboxyl group or its bioisostere. The structures of hit compounds 3 and 4 were confirmed by high resolution mass spectroscopy. Compound 3 is known as 3-(2-(4-Chloro-*N*-methylbenzamido)phenyl)propanoic acid (37) and compound 4 as (*E*)-3-(2-(*N*-Methyl-4-(methylthio) benzamido) phenyl)propenoic acid.

### Cell culture methods, plasmids

Cos-1 cells, ESC and MOVCAR were maintained as described^47^,^48^ in medium containing dextran-coated charcoal-treated fetal bovine serum. MOVCAR, A2780, KPC cell lines were gifts from Dr. Sundaram Ramikrishnan, University of Minnesota^49^,^50^. Plasmid transfection was conducted using Lipofectamine 3000 (Invitrogen). Mouse *Crabp1* cDNA was cloned into pCMX-PL1. Mutations of Crabp1 R131A, and Y133F were introduced using site directed mutagenesis (Stratagene).

### ESC generation

Crabp1 knockout ESC were established previously^51^, and used to establish Crabp1 knockout mice. Crabp1 KO ESC were derived from day 2.5 morula from Crabp1 KO mice as described^52^. All animal procedures have been approved and were performed according to the University of Minnesota IACUC Animal Care Committee guidelines.

### Western blotting and chemicals

Whole cell lysate was prepared as described^53^,^54^. Antibodies for β-actin (SC-47778), ERK1 (SC-93), and ERK2 (SC-153) were from Santa Cruz. Antibodies for Crabp1 (C1608), flag (F3165) were from Sigma. Anti-phospho-ERK1/2 (9101) and cleaved caspase-3 (9661) were from Cell Signaling. Anti-GST (05–311) was from Upstate. AtRA (100 nM) was from Sigma. AGN 193109 (RAR antagonist, 100 nM) was from Santa Cruz. 5-(2-phenyl-pyrazolo[1,5-a] pyridin-3-yl)-1H-pyrazolo[3,4-c]pyridazin-3-ylamine (ERK1/2 inhibitor, 1 μM) was from EMD. ^3^H-RA was from Perkin Elmer.

### *In vitro* ligand binding competition assay

*In vitro* ligand binding competition assay was as described^10^. Recombinant His-Crabp1 was purified from bacteria. In 300 μl of binding buffer (50 mM HEPES, pH 8.0, 100 mM NaCl, 1 mM EDTA, 10% glycerol, 0.1% Nonidet P-40), an equimolar concentration of Crabp1 (100 nM) and [^3^H]-atRA was incubated for 40 min at room temperature in the presence of excess cold ligand (1 μM). His-tagged Crabp1 was affinity-captured on nickel-nitrilotriacetic acid-agarose affinity beads (Qiagen) for 2 hrs at 4 °C, and washed twice with binding buffer. Ligand-bound Crabp1 was dispersed in scintillation mixtures, and the radioactivity was measured in a liquid scintillation counter (Beckman).

### *In vitro* ligand binding assay

His-Crabp1 (WT, R131A, and Y133F) proteins were purified from bacteria. In 300 μl of binding buffer (50 mM HEPES, 100 mM NaCl, 1 mM EDTA, 10% glycerol, 0.1% Nonidet P-40, pH 8.0), an equimolecular concentration of Crabp1 and [^3^H]RA was incubated for 40 min at room temperature. His-tagged Crabp1 was affinity-captured on nickel-nitrilotriacetic acid-agarose affinity beads (Qiagen) for 2 hrs at 4 °C, and washed twice with binding buffer. Ligand-bound Crabp1 was dispersed in scintillation mixtures, and the radioactivity was measured in a liquid scintillation counter (Beckman).

### *In vitro* kinase assay

Semi *in-vitro* kinase assay was performed as described^10^. Cos-1 cells expressing flag-Crabp1 were lysed and immunoprecipated^55^ with anti-flag antibody. The precipitated complex was incubated with pure 0.4 μg GST-ERK2 (14–198, Upstate) in 40 μl kinase buffer (20 mM MOPS pH 7.2, 25 mM β-glycerolphosphate, 5 mM EGTA, 1 mM Sodium orthovanadate, 1 mM DTT, 120 μM ATP, 18 mM MgCl_2_, 1X Protease inhibitor) at 30 °C for 30 min. Samples separated on SDS-PAGE gel were detected with anti-phospho-ERK1/2.

### Phosphatase assay

Serine/threonine phosphatase (V2460, Promega) and PP2A phosphatase (17–313, Upstate) assays were performed as manufacturer’s instructions. Reaction times were 10 min at 30 °C. ESC were treated with 100 nM atRA, C3, or C4 for indicated time points. Phosphatase activity was detected at optical density 630 nm in a microplate reader (Tecan M1000).

### Flow cytometry

After treatment of 100 nM for 12 hrs, cells were harvested and fixed with 70% ethanol overnight at −20 °C. Cells were then stained with staining buffer (1XPBS containing 100 μg/ml DNase-free RNase A and 40 μg/ml propidium iodide) at 37 °C for 30 min. Cells were analyzed with fluorescence-activated cell sorting (BD Accuri).

### MTT assay

Cell viability was assessed by measuring their ability to metabolize 3-(4,5-dimethyldiazol-2-yl)-2,5-diphenyltetrazolium bromide (MTT) using the TOX-1 kit (Sigma Aldrich) at 24 hr timepoint after 100 nM atRA and C3 treatment.

### Detection of apoptosis

Cell apoptosis was detected using CytoGLO pSIVA IANBD kit (IMG-6701K, IMGENEX) Images were acquired by Olympus FluoView 1000 IX2 upright confocal microscope. The fluorescence intensity representing pSIVA positive cell number from different fields was counted using ImageJ and quantified.

### Data analysis

Analyses of data were performed using appropriate analysis of variance. Significant effects were followed with appropriate post hoc tests. In all cases, statistic analyses were done by two-tailed Student’s *t*-test, and the comparison was considered statistically significant when *p* < 0.05. Data were presented as means ± S.E.M. All experiments used an N of three unless otherwise indicated.

## Additional Information

**How to cite this article**: Persaud, S. D. *et al*. All trans-retinoic acid analogs promote cancer cell apoptosis through non-genomic Crabp1 mediating ERK1/2 phosphorylation. *Sci. Rep*. **6**, 22396; doi: 10.1038/srep22396 (2016).
