# Corrigendum: All trans-retinoic acid analogs promote cancer cell apoptosis through non-genomic Crabp1 mediating ERK1/2 phosphorylation

**DOI:** 10.1038/srep27678

**Published:** 2016-07-20

**Authors:** Shawna D. Persaud, Sung Wook Park, Mari Ishigami-Yuasa, Naoko Koyano-Nakagawa, Hiroyuki Kagechika, Li-Na Wei

Scientific Reports 6: Article number: 2239610.1038/srep22396; published online: 03032016; updated: 07202016

This Article contains errors. In the last paragraph of the Results section,

“Given the ability of atRA and compounds 3 and 4 to induce apoptosis in animal cancer cell line context, we surveyed several human cell lines and found that CRABP1 expression was lost in human ovarian cancer cell line A2780 and human pancreatic ductal carcinoma cell line KPC”.

should read:

“Given the ability of atRA and compounds 3 and 4 to induce apoptosis in animal cancer cell line context, we surveyed several additional cell lines and found that CRABP1 expression was lost in human ovarian cancer cell line A2780 and mouse pancreatic ductal carcinoma cell line KPC”.

In addition, the legend of Figure 4D,

“(**D**) Compounds lose ability to induce ERK activity in human CRABP1 null cancer cell lines A2780 (ovarian) and KPC (pancreatic ductal carcinoma) after 100 nM, 30 min treatment (upper). Compounds are able to induce apoptosis after 24 hr treatment (lower) in CRABP1 positive MCF7 breast cancer cell line (lower right). Quantification of cleaved caspase 3 is shown for MCF-7. Data (**A**–**D**) are representative of at least 3 independent experiments”.

should read:

“(**D**) Compounds lose ability to induce ERK activity in human CRABP1 null cancer cell line A2780 (ovarian) and mouse KPC (pancreatic ductal carcinoma) after 100 nM, 30 min treatment (upper). Compounds are able to induce apoptosis after 24 hr treatment (lower) in CRABP1 positive MCF7 breast cancer cell line (lower right). Quantification of cleaved caspase 3 is shown for MCF-7. Data (**A**–**D**) are representative of at least 3 independent experiments”.

